# Data Measurement, Instruments and Sampling

**DOI:** 10.1111/jpm.13142

**Published:** 2024-12-10

**Authors:** Paul Slater, Felicity Hasson

**Affiliations:** ^1^ Institute of Nursing and Health Research Ulster University Coleraine UK

**Keywords:** data management, instruments, quantitative, sampling

## Abstract

Data measurement, instrument selection, and sampling are fundamental elements in quantitative research and data collection. Data measurement is the systematic assignment of numeric values or categories to variables to permit measurement with precision and accuracy. Instrument selection involves selecting the right tool or method to collect data effectively and accurately and ensure the reliability and validity of the data. Sampling is the process of selecting a subset of individuals or items from a larger population to best represent that population, free of bias and generalizable. Understanding these key terms ensures that data collected is valid, reliable, and appropriate for future statistical analysis and interpretation.

## 1 Introduction

Within this series, previous papers focus was on philosophical underpinning of quantitative research methods (Slater 2024) and major research designs (Slater and Hasson 2024) that help set a methodology context and align with the study research objectives (see Box 1). Building on this knowledge, this paper will help categorise and describe the methods and tools/instruments that are used in a study, sampling techniques and sample size. It is the interface between the methodologies and methods, which is important as this informs the data analysis. Then the process of selecting the appropriate statistical test to examine hypotheses is relatively straightforward. Before we outline considerations relating to sampling key, decisions need to be made relating to the time and variable type.

BOX 1 **Methodology Versus Methods.**

Methodology: Concerns the overall strategy (quantitative) used, including the theoretical framework and its rationale for use in addressing the research question. It is concerned with the ‘why’ and the rules of the ‘methodological framework’ of a research design.

Methods: Concerns the specific tools/instruments and procedures used to collect and analyse data. It is the ‘how’ data are collected and analysed within the methodology framework.

A well‐defined methodology helps ensure that the methods are rigorously and correctly selected and applied, enhancing the study's reliability, validity and ethical soundness.

### 1.1 Time Scales in Research

**FIGURE 1:**
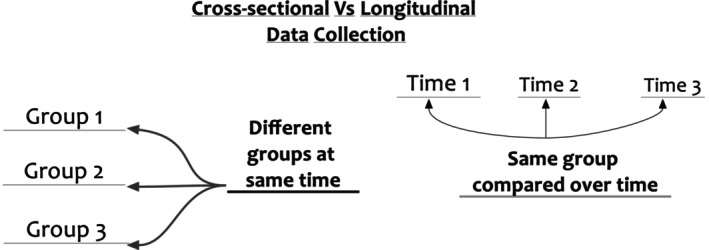
Cross‐sectional versus longitudinal data collection.

Time frame is an important consideration in any research design as it will influence the type of questions asked, as well as the survey design. There are two main types of studies: cross‐sectional and longitudinal. The difference between them is the number of time data are collected (see Figure 1).

#### 1.1.1 Cross‐Sectional Design

Data are collected at one time point, with participants only providing data for that timepoint. It is a form of observational design where both the outcomes (dependent) and exposures (independent) variables of the participants are measured at the same time (see Figure 1). It is most associated with the research design that shares its name—the cross‐sectional survey design. It is quick and cost‐effective and provides a snapshot of the population. As a result, it has limited internal validity, such as causal and temporal inference and prone to sampling bias.

#### 1.1.2 Longitudinal Repeated Measures Design

This is when data are collected from the same participants on two or more occasions and the participants' responses are paired across timepoints (see Figure 1). It means that change can been measured across time and the respondents baseline scores may be used as their own control (within‐subject design; see Box 2). This is associated with experimental research designs such as randomised controlled trials when data are collected from the same individuals before and after an intervention has taken place. It requires smaller sample sizes and allows for better understanding for causal and temporal inferences (internal validity).

BOX 2 **Sample Calculator.**

For a fuller account of sample size calculation see: McCrum‐Gardner (2010).

Free sample size calculator computer software.

https://www.calculator.net/sample‐size‐calculator.html.

https://clincalc.com/stats/samplesize.aspx.

### 1.2 Variable Type

A variable is a measure that may be assigned different values at different times and/or for different participants, for example, height, clinical disorder or reaction time. In most experiments we are interested in the effect of one variable on another: for example, the effect of alcohol consumption on reaction time. The variable causing the change is the independent variable and the one which is changed in the dependent. The experimenter aims to manipulate the independent variable (IV); for example, whether a person receives and intervention or not and measure its impact on the dependent variable (DV) whether it has an impact on clinical recovery. Choosing which variables to measure is central to good experimental designs.

#### 1.2.1 Independent Variables

The independent variable is the cause. It is the variable we, as researchers, directly manipulate to measure its effect (or not) on another variable. There are two main types, experimental independent variables can be directly manipulated by the researcher such as assignment to intervention or control group. This variable is manipulated to see how they affect your dependent variable. An independent variable can be a treatment such as a drug dosage or a risk factor such as diet. While subject independent variables cannot be manipulated and relate to characteristics of participants that cannot be changed such as age or gender identity, ethnicity, race and education, which can be used to group research subjects categorically.

#### 1.2.2 Dependent Variables

The dependent variable of (criterion variable) is the variable that the researcher is interested in understanding, explaining or predicting. It is the variable that changes because of the independent variable manipulation, that we aim to influence when examining the effectiveness of an intervention. For example, a dependent variable may be depression symptoms, which is depends on the independent variable (type of therapy provided). The label of dependent or independent is not static on a variable for example, a variable may be the dependent in one hypothesis and be the independent in another test. Dependent variables are also called response (they respond to change in another variable), outcome (they represent the outcome you intend to measure) and life‐hand‐side variables (they appear on the left‐hand side of a regression equation).

## 2 Measurements and Instruments

### 2.1 Levels of Measurement

The fundamental tenet of quantitative research is the measurement, and there are multiple categories of type (or scales) and methods of measurement. Your type of measurement will influence how you analyse your data. There are four main types of measurement: nominal, ordinal, interval and ratio.

#### 2.1.1 Nominal Data

In quantitative research, some variables can be allocated scores to help classify them as belong to one category or another. There is no hierarchy and the code allocated to completely arbitrary and for categorisation purposes only. For example, the presence of absence of depression or whether a participant is a mental health nurse or an adult nurse. These are called nominal variables—in name only (Parahoo 2014).

#### 2.1.2 Ordinal Data

Ordinal data measurements allow us to imply an ordering or rank of the categories collected; for example, never, sometimes and always. In this rank ordering we can legitimately say that the item ranked first is better than second, but we cannot determine the magnitude of the difference. It is the unknown or changeable nature of the relationship between categories that identifies it as ordinal data and distinguishes it from interval; it is the rank ordering that distinguishes it from nominal. For example, frequency of engaging in physical exercise can be categorised into the following rankings, never, rarely, sometimes, often and always. This illustrates a clear order of the categories.

#### 2.1.3 Interval

An interval scale is a measurement scale with an equal distance between values but no true zero point. For example, weight, where a change between 3 and 4 kg is the same (1 kg) as between 110 and 111 kg. The best example of both types of measurement is temperature: for example, Celsius/Fahrenheit where temperatures can extend into minus numbers, yet in Kelvin there is an absolute zero.

#### 2.1.4 Ratio

Like interval level of measurement this scale has equal distance. While it shares features with interval data, a distinguishing feature is that this type of data can have a value of absolute zero. Examples of ratio data include income, height, weight, crime rate and age.

### 2.2 Measurement Types

Methods of measurement refer to survey scales, which provide orderly arrangement of survey response options. They can be presented verbally or numerically from which respondents indicate their level of feeling about the measured attribute. Several types of measurement exist, including, dichotomous scales, Likert, visual analogue and semantic differential scales.

### 2.3 Dichotomous Questions

These are nominal in nature and provide two (or more) options, which lie at opposite ends, such as yes/no, trust/false or present/absence. They type of response option does not provide the respondent with a neutral answer, therefore is used when you need to gather precise data.


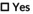
 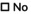


### 2.4 Likert Scales

In quantitative research, the most used rating scale is a Likert scale. A Likert scale measures behaviours, attitudes or opinions. It assumes there is a linear relationship between the categories on a particular measure and respondents are asked to identify where they fall along that linear continuum. Likert scales commonly measure along five or seven options (e.g., strongly disagree, neither agree nor disagree, and strongly agree) and they allow researchers to examine more nuanced views on a subject area rather than binary views of ‘agree’ or ‘disagree’. The numerical conversion of the response would be considered as an ordinal or interval scale.


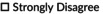
 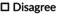 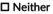 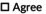 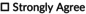


### 2.5 Visual Analogue Scale

A visual analogue scale (VAS) tries to measure a characteristic or attitude across a straight line with the ends anchored at two extremities such as pain or fatigue. The respondents mark their perspective on the line between the two extremes. The numerical conversion of the response would be considered as an interval scale.


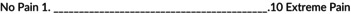


### 2.6 Semantic Differential Scales

These are less frequently used in recent research but still useful in exploring a concept or phenomenon. It comprises of three parts: stem, anchors and steps. The stem is the overarching construct being examined. Elements of the series of adjectives related to the stem are posed as adjectives and opposing autonyms (anchors) posed at opposite ends of a continuum (steps). Respondents are asked to mark along the continuum where they feelings relating to the stem and anchor words rest. The numerical conversion of the response would be considered as an interval scale.


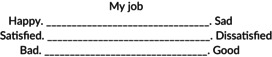


### 2.7 Types of Instruments

Quantitative data can be collected using a variety of methods and the main types are identified in Table 1. These methods of data collection can be conducted using both cross‐sectional and longitudinal research designs.

**TABLE 1:** Data collection methods in quantitative research.

Type	Description
Questionnaire	Surveys are the most used form of data collection and can be used on a wide range of research topics. Surveys can be conducted as self‐report, online, in person or over the phone. They are usually designed to collect specific information relating to beliefs, attitudes and behaviours. Surveys usually contain closed‐end questions with a defined range of answer options available to select. Standardised diagnostic tools: A good survey will have been rigorously developed and with proven psychometric properties aimed to address specific psychological conditions such as depression or anxiety. For example, Beck Anxiety Inventory Beck et al. (1988). Knowledge scales: These are tests specifically design to measure a respondent's knowledge on a particular area, such as dementia knowledge or diabetes. Measured on a correct/incorrect binary classification that are cumulatively scored to give an overall measure. For example, Dementia Knowledge Assessment Scale Annear et al. (2015). Functional ability scales: These are tools used to evaluate a person's ability to perform activities of daily living and/or instrumental activities of daily living. For example, Barthel Index Mahoney and Barthel (1965). Self‐developed prevalence tools: Usually used in descriptive exploratory cross‐sectional designs when little knowledge and no measurement tools exist to measure the subject area. The findings provide a descriptive account of the subject area.
Observations	Unstructured or structured observations can be collected within natural settings. Unstructured observations tend to be sporadic and recorded in words. In structured observations the researcher clearly specifies boundaries such as what to observe and length of time to conduct the observations. The observations are quantified for further analysis. The drawback is that observations are subjective. Observations straddle the link between qualitative and quantitative research. For example, WHO Surgical Safety Checklist World Health Organization (2009) or the Facial Action Coding System.
Bio‐physiological measures	These measures are usually collected by self‐report or specialist equipment such as sphygmomanometer for collecting blood pressure. When using electronic equipment, the findings tend to be more accurate (if appropriately calibrated beforehand). For example, blood pressure; heart rate, respiration and skin temperature.
Document review/secondary data	Data can be extracted from existing sources such as educational records, medical notes or government research. This has become a growing trend in recent times due to big data linkage. The records are usually recorded for reasons other than research and consequently may vary in quality.
Computerised tracking	Digital data can be collected using electronic sources such as eye‐tracking movement software or heatmapping. It presents clear and unequivocal data, for example, GPS tracking software.

The selection of the right measurement tool is very much dependent on the aim of the study, the variables of interest, the population and how you intend to access your sample. Where possible, ensure the instrument(s) is appropriate for the sample and has proven measures of validity and reliability. These can be inferred from research literature findings and good research should, where possible, generate their own measures of validity and reliability but this is not always possible.

## 3 Sampling

Samples and sampling are fundamental elements the help determine the internal and external validity of a research study findings. A sample is a specific group of individuals drawn from the population to participant in the study, while sampling technique is the process used to select the participants. The selection of a sampling technique that maximises representativeness of the sample to the population under investigation helps to minimise selection bias and improve the internal validity of a study (Slater and Hasson 2024).

### 3.1 Population/Sampling

A population is the total number of units (often people) from which the data can potentially be collected (Parahoo 2014). The units are defined by the inclusion criteria. In research, we often cannot not collect information from an entire population due to various reasons such as financial costs, access or ethics. Instead, we choose to select a sample that we can access and that we try our best to ensure it will be indicative of the wider population—if it is, then the samples views will reflect the populations.

#### 3.1.1 Inclusion/Exclusion Criteria

Inclusion criteria are the specific characteristics or attributes that participants must have to be eligible to participate in a study. They define the population and ensure that the research focuses on the appropriate group to address the study objectives. A sampling frame refers to the actual list that (i.e., employee database) that the sample will be drawn from a clearly defined inclusion and exclusion criteria sets the parameters for the population and sampling frame and identifies who exactly the results are generalisable to as well as help with the sample size calculation of required respondents. Key elements of inclusion criteria in mental health nursing include age, condition status, language or cognitive limitations.

Each sampling method has unique strengths and weaknesses, challenges and opportunities. The selection of the this must be balanced with the study objectives and what is achievable.

Sampling strategies vary; however, there are two types of sampling: probability and non‐probability. Probability sampling such as randomised sampling is a sample selection where everyone has an even chance of being selected. Non‐probability sampling techniques are where the researchers select the sample based on non‐random factors. Selection of either method depends on the nature of the study.

There are several types of probability and non‐probability sampling methods you can employ (see Table 2).

**TABLE 2:** Sampling techniques.

Sampling technique	Description	Advantages	Limitations
Probability sampling			
Simple random sampling	Each participant is chosen randomly from the sampling frame, with every person in the sampling frame having an equal chance of being selected.	Reduces selection bias, highly generalisable.	Requires a complete sampling frame.
Systematic sampling	Selecting every nth individual from the sampling frame after determining the interval needed to reach the sample size.	Easy to implement, efficient for larger samples.	Patterns in the sampling frame can introduce bias, for example, surnames.
Cluster sampling	Dividing the population into clusters and randomly selecting entire clusters to make up the sample. For example, hospital units or clinics.	Cost‐effective for large populations and mitigates for Hawthorne effect or contamination.	High within cluster homogeneity can reduce variability.
Stratified sampling	Dividing the sampling frame by specific criteria (e.g., age) and randomly sampling within each subgroup to ensure diversity and representation.	Ensures all subgroups are represented.	Complex to organise and more criterion related knowledge of the participants; may require a larger sample size.
Non‐probability sampling			
Convenience sampling	Where the sample is taken from a group of people close at hand or easily contacted. There are no other criteria than they are willing to participate and close at hand.	Quick, easy and convenient to collect data.	High risk of bias, limits generalisability.
Purposive sampling	Participants are selected due to the qualities they possess. The judgement of the researcher helps select the participants, based on a clear inclusion criterion.	Focuses on specific expertise or experience.	Results may not apply to the general population.
Consecutive sampling	Every subject meeting the inclusion criteria is selected until the sample size is achieved. This may be over a defined period of time.	Captures data over time, practical in clinics.	Sample may be biased by external events and/or limited to a period of time.
Quota sampling	You select a set number of participants from predetermined groups such as age, gender, or ethnic groups so that all views are actively represented in the overall sample.	Ensures all subgroups are effectively represented.	Selection bias if quotas are based on convenience.

Care and consideration must go into the selection of the most appropriate sampling technique for a study as it impacts on the quality, generalisability and meaningfulness of the findings. The next challenge is to determine how many people to select.

### 3.2 Sample Size Calculation

A sample size calculation is helps determine the number of participants you need to bother! In many quantitative, the inclusion of a sample size calculation is an essential requirement for ethical approval, grant funding or publication of findings (McCrum‐Gardner 2010). Determining an appropriate sample size is essential in research because it ensures that the study has enough power to detect significant effects without making the study unnecessarily unwieldy, unethical or expensive. Statistical software packages are freely available that help with the computation of a required sample size (see Box 2). However, there are several key factors that impact on a sample size calculation and these need to be determined before conducting a sample size calculation. These include significance level, power, effect size, research design, population size and variability. For more details, please see Box 2.

## Ethics Statement

The authors have nothing to report.

## Conflicts of Interest

The authors declare no conflicts of interest.

## Data Availability

The authors have nothing to report.
